# Data sets of human and mouse protein kinase inhibitors with curated activity data including covalent inhibitors

**DOI:** 10.2144/fsoa-2023-0088

**Published:** 2023-08-16

**Authors:** Elena Xerxa, Jürgen Bajorath

**Affiliations:** 1 Department of Life Science Informatics, B-IT, LIMES Program Unit Chemical Biology & Medicinal Chemistry, Rheinische Friedrich-Wilhelms-Universität, Friedrich-Hirzebruch-Allee 5/6, D-53113 Bonn, Germany

**Keywords:** activity data, analogue series, covalent inhibition, inhibitors, large-scale data analysis, protein kinases

## Abstract

**Aim:**

Generation of high-quality data sets of protein kinase inhibitors (PKIs).

**Methodology:**

Publicly available PKIs with reliable activity data were curated. PKIs with very weak activity were classified as inactive. Analogue series and PKIs containing reactive groups (warheads) enabling covalent inhibition were systematically identified.

**Exemplary results & data:**

A total of 155,579 human and 3057 mouse PKIs were obtained. Human PKIs were active 440 kinases and included 13,949 covalent PKIs. The collection of qualifying PKIs and corresponding inactive compounds is made available as an open access deposition.

**Limitations & next steps:**

Potential limitations include activity data incompleteness and assay variance. The data set can be used to investigate PKIs with alternative modes of action and calibrate computational methods.

## Plain language summary

Protein kinases are proteins that play a role in how cells grow. In cancer cells, protein kinases are altered, which can cause abnormal growth. Protein kinase inhibitors (PKIs) specifically target protein kinases and are considered for treating different diseases, like cancer. In this study, we investigated a large number of PKIs that are available to the public to find ones with reliable activity data. We aim to understand how their structure affects their activity, including how these compounds bind to protein kinases. This helps us to identify different types of PKIs. Understanding PKIs is important for both basic research in the protein kinase field and drug discovery.

## Graphical abstract

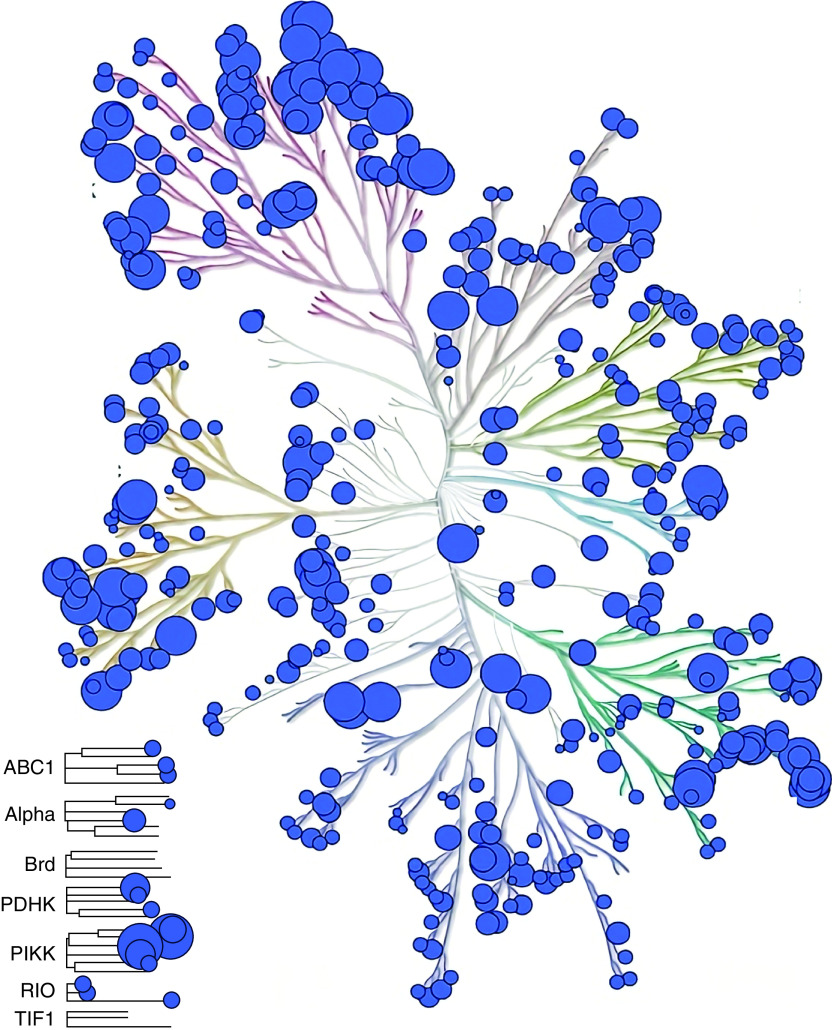

In drug discovery, inhibitors of human kinases are intensively investigated for a variety of therapeutic applications. Shown is a phylogenetic tree representation of the human kinome. Blue dots represent protein kinases for which chemical inhibitors are available. The dots are scaled in size according to the number of inhibitors per kinase. Currently available inhibitors cover more than 80% of the human kinome.

PKIs are intensely investigated as drug candidates in different therapeutic areas [1–3]. The human kinome comprises 518 kinases [4]. Currently, PKIs with high-confidence activity data are available for more than 400 kinases, as further discussed below, and most of these extensively or still preliminarily explored kinases are considered as drug targets for the treatment of a variety of acute or chronic diseases [1–3]. Thus far, 71 human PKIs are approved as drugs by the US FDA [5]. Most PKIs are directed against the adenosine triphosphate (ATP) cofactor binding site in the catalytic kinase domain that is essentially conserved across the human kinome, giving rise to frequent multi-kinase activity of PKIs and polypharmacology [2,3]. ATP site-directed PKIs mostly act by competitive binding including different inhibitory mechanisms [6,7]. These PKIs either occupy the ATP pocket or bind to the activation loop and stabilize an inactive conformation. Other PKIs elicit allosteric effects at various sites distributed across the catalytic kinase domain [6,8] or covalently inhibit kinases [9,10]. Competitive or allosteric covalent inhibition is facilitated by targeting the side chains of free cysteines or other residues with reactive groups conventionally termed warheads [9–11]. Compared with competitive ATP site-directed PKIs, only limited numbers of covalent and especially allosteric PKIs have been reported thus far.

In recent years, there has been substantial growth in the number of PKIs that are publicly available [12,13]. To our knowledge, the last comprehensive survey of public domain PKIs was reported in 2018 [13]. Publicly available PKIs provide a large knowledgebase for basic research and drug discovery. Five years after the last survey, we have systematically curated PKIs for which reliable activity data are available and generated large data sets for follow-up analysis. These data sets and an open access deposition making them freely available are described herein.

## Methodology

### Data curation

Protein kinase information was retrieved from UniProtKB/Swiss-Prot (release 3 August 2022) [14]. A list of unique identifiers (Uniprot IDs) of human and mouse PKs was used to screen the ChEMBL (version 31) [15] and BindingDB [16] databases for PKI activity data (both databases were accessed in October 2022).

ChEMBL: Only PKI activity annotations falling into the SINGLE PROTEIN target category tested in direct interaction assays at the highest confidence level (ChEMBL confidence score 9) with standard activity measurements (IC_50_, K_i_, K_d_) in nanomolar (nM) units were considered. Activity values were recorded in negative decadic logarithmic form. Compounds with problematic activity comments including ‘uncertain’, ‘potential transcription error’, and ‘outside typical range’ or inconsistent ‘active’ / ‘inactive’ labels were omitted. Furthermore, a threshold of 10,000 nM was applied for classifying a PKI as active. Compounds with reported lower activity (that is, borderline active PKIs or PKIs with questionable activity) were classified as inactive.

BindingDB: PKIs were extracted on the basis of UniProt IDs for human and mouse protein kinases. Standard activity measurements including IC_50_, K_i_, and K_d_ values with activity relationship “=” reported for single chain targets were considered also applying the 10,000 nM threshold for inhibitory activity. Compounds with activity relationship “>” and standard values of at least 10,000 as well as PKIs with activity relationship “=” and standard values larger than 10,000 nM were classified as inactive.

SMILES strings [17] representing qualifying PKIs from ChEMBL or BindingDB were subjected to a standardization protocol that included canonicalization, neutralization, removal of salts and stereochemical information. PKIs from ChEMBL and BindingDB were combined using the resulting unique non-stereo sensitive canonical SMILES strings.

If multiple qualifying potency measurements (standard activity relation “=”) of the same type were available for a kinase-inhibitor pair, the mean was calculated as the final potency annotation. If IC_50_, K_i_, and/or K_d_ value were available, assay-independent K_i_ or K_d_ were selected. Activity annotations calculated from multiple values were discarded if the standard deviation was greater than 1 (logarithmic units). Kinase-inhibitor pairs for which conflicting quantitative and qualitative measures (standard activity relation “>” or “>>”) were available in ChEMBL and/or BindingDB were also disregarded.

**Figure 1.**
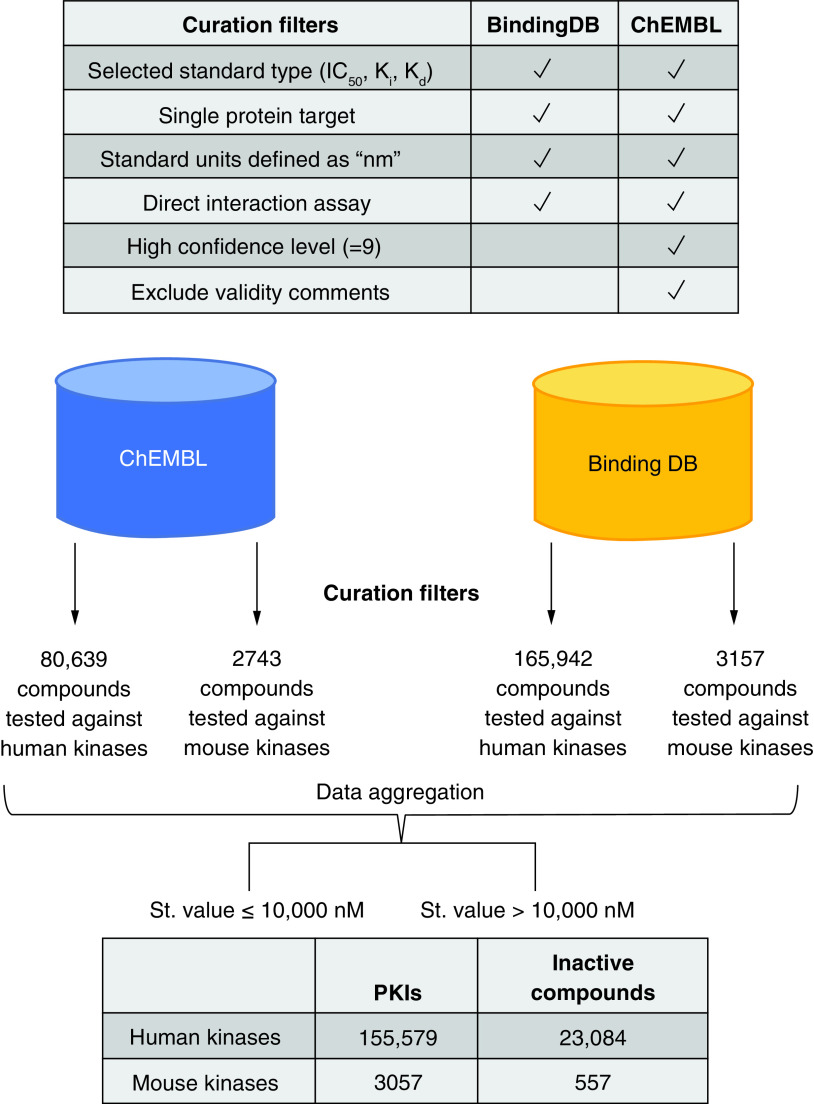
Data curation and aggregation. The workflow diagram summarizes protein kinase inhibitor data curation and aggregation steps described in the text. PKI: Protein kinase inhibitor.

Finally, the selected compounds were organized in separate data sets of active human and mouse PKIs and corresponding compounds classified as inactive. Figure 1 summarizes the PKI data curation and aggregation process.

### Analogue series

**Figure 2.**
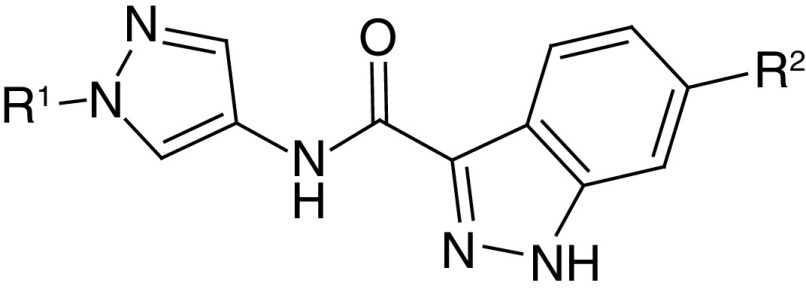
Exemplary analogue series. Shown is a core structure-based representation of an analogue series with activity against the Aurora kinase family. The core structure is shared by multiple analogues forming this series that are distinguished from each other by different combinations of R-groups at two substitution sites (designated R^1^ and R^2^, respectively).

An analogue series (AS) consists of several compounds that share the same core structure (also termed scaffold) and are distinguished by different substituents (R-groups) at one or more sites, as illustrated in Figure 2. From human and mouse PKIs, AS with single or multiple substitution sites were systematically extracted using the compound-core relationship (CCR) algorithm [18]. The CCR approach systematically fragments compounds based on retrosynthetic rules and identifies all compound core structures after removal of substituents. All possible compound–core relationships are explored and compounds containing the same core are assigned to an AS. Hence, each AS represents a unique core structure.

### Covalent inhibitors

To identify covalent PKIs, substructure searches were carried out in the human PKI data set with 14 commonly used warheads targeting the side chains of different amino acid residues [19].

## Exemplary results

### Protein kinase inhibitors

**Figure 3.**
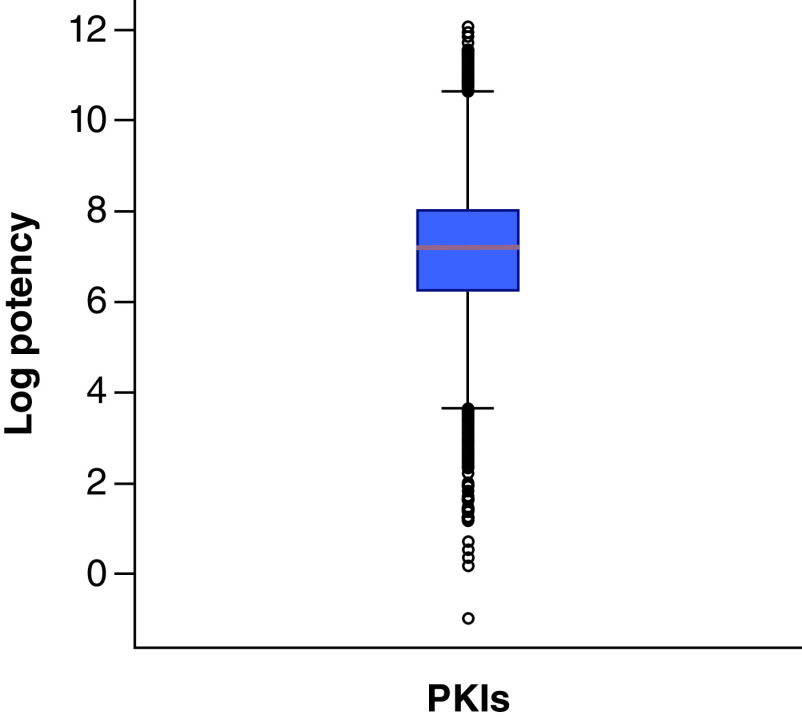
Potency of human protein kinase inhibitors. The boxplot represents the potency value distribution of all qualifying human protein kinase inhibitors. In a boxplot, the value distribution is represented by its minimum (lower whisker), lower quartile (lower boundary of the box), median (horizontal line in box), upper quartile (upper boundary of the box) and maximum (upper whisker). Diamond symbols represent values classified as statistical outliers. PKI: Protein kinase inhibitors.

A total of 155,579 qualifying unique human PKIs were obtained and a comparably small set of 3057 mouse PKIs. Human and mouse PKIs formed a total of 237,620 and 3685 unique compound-kinase interactions, respectively. Human PKIs were active against 440 kinases, providing ∼85% coverage of the human kinome, and mouse PKIs were active against 109 kinases (that is, each PKI inhibited one or more kinases). In addition, 14,240 compounds were classified as inactive (inhibitory activity >10,000 nM) against 343 human kinases and 481 compounds as inactive against 39 mouse kinases. Compared with a previous survey reported in 2018 [13], the number of human PKIs with reliable activity data has further increased by ∼43,000 compounds. Figure 3 shows the logarithmic potency distribution of the human PKIs, with a median potency value of 7.1 (corresponding to ∼100 nM potency). Most PKIs were active in the logarithmic potency range of 6–8. We have used the new data set of human PKIs to analyze the promiscuity and potency distribution of different types of PKIs, among other properties, and identify activity cliffs formed by PKIs and potential covalent inhibitors, as reported recently [20].

### Analogue series & core structures

From human PKIs, 29,298 AS were algorithmically extracted and 41,171 singletons were identified (that is, PKIs having no structural analogues), as reported [20]. Mouse PKIs yielded 714 AS and 856 singletons. Because each AS and each singleton contained a unique core structure, human and mouse PKIs yielded a total of 70,469 and 1570 distinct cores, respectively. Although algorithmically defined core structures are often similar (yet distinct), core structure diversity among PKIs is generally high [20].

### Covalent inhibitors of human kinases

**Figure 4.**
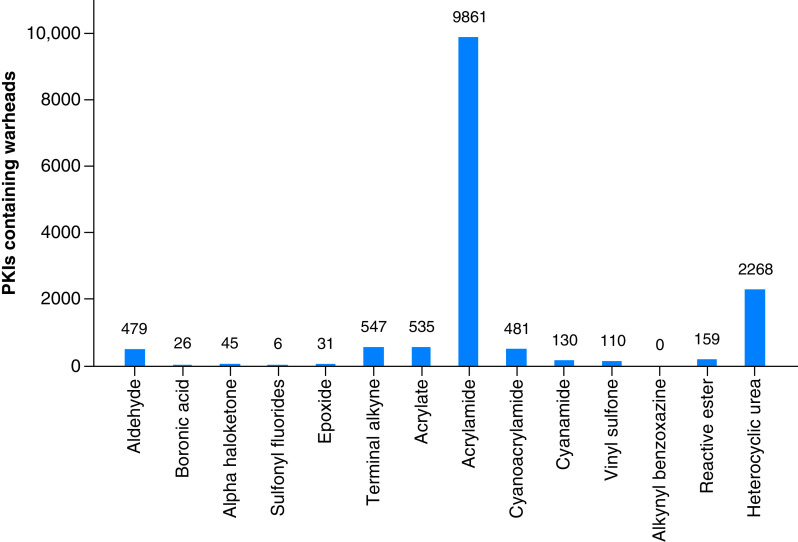
Covalent human protein kinase inhibitors. The histogram shows the distribution of human protein kinase inhibitors containing 14 chemical warheads enabling covalent inhibition. PKI: Protein kinase inhibitor.

CovalentInDB, a database for covalent inhibitors [21], currently contains ∼2000 PKIs. We searched our human PKI dataset for compounds with 14 different warheads and identified 13,949 potential covalent PKIs. Figure 4 shows the distribution of compounds over these warheads. Acrylamide (targeting Cys, Lys, Ser and Thr residues) and heterocyclic urea (targeting Ser and Thr residues) were the most frequently detected warheads in PKIs, with 9861 and 2268 instances, respectively. The median potency values of all warhead-dependent subgroups of covalent PKIs for which at least 100 compounds were available fell into the logarithmic potency range of 6–8. Overall, an unexpectedly large number of potential covalent PKIs was identified, providing ample opportunities for follow-up analysis.

## Data

Newly curated human and mouse PKIs and corresponding compounds classified as inactive were organized in four ‘tab separated values’ (tsv) files containing the following information:

- Compound_new_ID: internal ID assigned to each compound,
- nonstereo_aromatic_smile: standardized compound SMILES,
- Uniprot_ID: UniProtKB/Swiss-Prot human and mouse kinase IDs,
- pref_name: preferred/full name of the kinase family,
- activity_id: reference to the activity data from ChEMBL and/or BindingDB,
- mean_log: mean negative logarithmic potency value (NaN: qualitative measurement),
- selected_stvalue: standard value used,
- ORGANISM: organism of the kinase,
- Human PKIs (human_PKI_active.tsv) containing warheads were flagged: CPKI: True.

These data sets and a readme.txt document have been made freely available on the ZENODO open access platform [22].

## Limitations & next steps

Importantly, for all human and mouse PKIs and corresponding compounds classified as inactive reported herein, high-confidence activity data are available. However, the resulting PKI activity profiles are generally affected by data incompleteness because PKIs have typically not been tested against the entire kinome (which especially applies to PKIs reported in older publications). Data incompleteness might lead, for example, to an underestimation of PKI promiscuity on the basis of currently available activity measurements. Furthermore, PKIs can be tested in many different assay formats, which might give rise to experimental heterogeneity and variance, especially for assay-dependent IC_50_ values. During data curation, we have addressed this issues by requiring highest assay confidence and by assessing the consistency of multiple activity measurements, if available. Data incompleteness and potential experimental heterogeneity are the only intrinsic PKI data limitations we currently are aware of.

The large number of newly curated human PKIs provides an extensive knowledgebase for the kinase field and a sound basis for follow-up investigations. For example, given the many AS formed by PKIs, these compound series yield a wealth of SAR information for medicinal chemistry. Furthermore, the significant number of putative covalent PKIs we identified enables the assessment of covalent inhibition and warhead characteristics on an unprecedentedly large scale.

Notably, from a medicinal chemistry point of view, PKI candidates with 10,000 nM activity are essentially regarded as irrelevant for drug discovery. However, both PKIs classified as inactive or active provide immediate opportunities for computational investigations. For example, they can be used for evaluating or calibrating potency prediction methods including machine learning regression models. Hence, data sets of inactive PKIs should also be useful for various applications.

**Summary points**

- Protein kinase drug discovery is introduced.
- Differences between protein kinase inhibitors (PKIs) are discussed.

**Methodology**

- Data curation is detailed.
- Identification of analogue series and covalent PKIs is described.

**Exemplary results**

- PKI statistics are provided.
- The potency distribution is assessed.
- PKI core structures and warheads are analyzed.

**Data**

- Data sets are described.
- The open access data deposition is detailed.

**Limitations & next steps**

- Data incompleteness and assay variance are discussed.
- Large-scale exploration of structure-activity relationships and covalent inhibition is enabled.
- Opportunities for computational investigations exist.
